# Discriminating Children with Speech Sound Disorders from Children with Typically Developing Speech Using the Motor Speech Hierarchy Probe Words: A Preliminary Analysis of Mandibular Control

**DOI:** 10.3390/diagnostics15141793

**Published:** 2025-07-16

**Authors:** Linda Orton, Richard Palmer, Roslyn Ward, Petra Helmholz, Geoffrey R. Strauss, Paul Davey, Neville W. Hennessey

**Affiliations:** 1 School of Allied Health, Curtin University, Perth, WA 6845, Australia; linda.orton@postgrad.curtin.edu.au (L.O.); g.strauss@curtin.edu.au (G.R.S.); p.davey@curtin.edu.au (P.D.); n.hennessey@curtin.edu.au (N.W.H.); 2 School of Earth and Planetary Sciences, Curtin University, Perth, WA 6845, Australia; r.l.palmer@curtin.edu.au (R.P.); petra.helmholz@curtin.edu.au (P.H.)

**Keywords:** speech sound disorders, assessment, kinematic, digital biomarkers, Motor Speech Hierarchy Probe Words

## Abstract

**Background/Objectives**: The Motor Speech Hierarchy (MSH) Probe Words (PWs) have yet to be validated as effective in discriminating between children with impaired and children with typically developing speech motor control. This preliminary study first examined the effectiveness of the mandibular control subtest of the MSH-PWs in distinguishing between typically developing (TD) and speech sound-disordered (SSD) children aged between 3 years 0 months and 3 years 6 months. Secondly, we compared automatically derived kinematic measures of jaw range and control with MSH-PW consensus scoring to assist in identifying deficits in mandibular control. **Methods**: Forty-one children with TD speech and 13 with SSD produced the 10 words of the mandibular stage of the MSH-PWs. A consensus team of speech pathologists observed video recordings of the words to score motor speech control and phonetic accuracy, as detailed in the MSH-PW scoring criteria. Specific measures of jaw and lip movements during speech were also extracted to derive the objective measurements, with agreement between the perceptual and objective measures of jaw range and jaw control evaluated. **Results**: A significant difference between TD and SSD groups was found for jaw range (*p* = 0.006), voicing transitions (*p* = 0.004) and total mandibular scores (*p* = 0.015). SSD and TD group discrimination was significant (at alpha = 0.01) with a balanced classification accuracy of 0.79. Initial analysis indicates objective kinematic measures using facial tracking show good agreement with perceptual judgements of jaw range and jaw control. **Conclusions**: The preliminary data indicate the MSH-PWs can discriminate TD speech from SSD at the level of mandibular control and can be used by clinicians to assess motor speech control. Further investigation of objective measures to support perceptual scoring is indicated.

## 1. Introduction

Speech sound disorder (SSD) refers to difficulties producing and using speech sounds and speech segments, resulting in reduced accuracy and clarity of speech production. It is the most prevalent of all childhood communication difficulties [1], affecting 3.4–5.6% of pre-school-aged children [2] and comprising more than 70% of a Speech–Language Pathologist’s (S-LP) caseload [3]. Children with SSD are more likely to experience adverse social, educational and psychological outcomes than children without SSD [4,5]. These difficulties may further limit employment opportunities throughout their lifespan [6]. Minimizing the impact of SSD is contingent on providing an accurate diagnosis to direct intervention approaches.

The causes of SSD can be organic or functional; organic SSDs arise from an underlying structural (e.g., cleft palate), motor/neurological or sensory/perceptual cause, while functional SSDs, which are more prevalent [5], are idiopathic and include articulation and phonological disorders [7]. Diagnosis of functional SSDs seeks to identify the underlying contribution of speech difficulties, for example, identifying whether the child is having difficulties learning the linguistic–phonological rules of the target language (i.e., a phonological impairment) and/or difficulties with the motor aspects of speech production. The differential diagnosis of SSD, however, is challenging due to the nature of SSDs and the limitations of current clinical practices [8,9,10]. McCabe, Korkalainen and Thomas [9] highlight that the “overlap between the symptoms of different disorders with the same speech features … from multiple different breakdowns…” complicates SSD, while Littlejohn and Maas [8] note that diagnosis is impacted by a “poor understanding of, and limited focus on the underlying impairment(s)” (p. 2).

As part of the assessment process, S-LPs are encouraged to conduct a comprehensive case history; an assessment of oral structures and hearing function; and an error analysis from a connected speech sample to identify a child’s phonetic inventory and phonological error patterns or processes, as well as obtain measures of the phonological mean length of utterance and quantify speech intelligibility [11]. In practice, however, time and ease of use are key factors influencing clinical decisions [12], with S-LPs routinely using standardized naming tasks to evaluate speech sound inventory and error patterns [12,13]. Measures of phoneme accuracy within the single-word naming tests, including the percentage of consonants correct (PCC), the percentage of vowels correct (PVC) and the percentage of phonemes correct (PPC), are frequently used to determine the presence and severity of SSDs [14].

The determination of speech sound error patterns typically relies on auditory–perceptual analysis using the International Phonetic Alphabet (IPA) transcription [14,15]. While this is a fundamental part of diagnosis, using auditory–perceptual assessment alone is limiting [16,17,18], and there is no gold standard validation of perceptual measures that can discriminate SSD from TD speech. Auditory perceptual assessments do not allow clinicians to determine the contribution of speech motor control to the production difficulties of a child [19]. Further, the reliance on assessment tools framed predominantly on linguistic models of speech production is problematic for differential diagnosis of SSD because these tools tend to focus on investigating phonological deficits [20], as opposed to the speech movement patterns associated with underlying constraints at the level of speech motor control.

McCauley and Strand’s [21] 2008 review of standardized tests that evaluate the speech motor performance of children concluded that “clinicians are in the position of having no tests that can be considered well developed for use with children with motor speech disorders” (p. 89). While new standardized assessment tools have been developed since this review, for example, the Dynamic Evaluation of Motor Speech Skill (DEMSS) [22], a recent review of assessment and intervention approaches for SSD identified 37 published assessment tools for SSD, with the majority focusing on specific skills and only four assessing combined articulatory, phonetic and motor-based development [4]. In their 2024 review of tools and approaches supporting the diagnosis of childhood motor speech disorders, McCabe, Korkalainen and Thomas [9] state, “there are not yet validated tools for comprehensively assessing all speech production processes” (p. 9).

A tool recently developed to measure speech motor control is the Motor Speech Hierarchy–Probe Words (MSH-PWs) [23]. The MSH [23] comprises seven stages that reflect the hierarchical (i.e., increasing levels of motor complexity) and interactive development of speech motor control: tone (Stage I), phonatory control (Stage II), mandibular control (Stage III), labial–facial control (Stage IV), lingual control (Stage V), sequenced movements (Stage VI) and prosody (Stage VII). The Probe Words (PWs) cover Stages III to VI, with 10 words and one phrase in each stage. The PWs were selected to reflect the primary articulatory movements required for word production at that stage of developing speech motor control [23]. Each PW is scored visually and auditorily by observing a child say the target word and judging whether the speech movements look and sound appropriate or inappropriate based on criteria outlined in the MSH-PW manual. For example, the criteria for mandibular control focus on assessing vertical jaw movements (e.g., through words containing bilabial sounds, mid-low vowels and simple syllable structures, like *map*, *bob* and *papa*) and how jaw control integrates with voicing and nasalization. These criteria include three measures of mandibular control—appropriate jaw range, appropriate jaw control stability and appropriate close–open phase—and two measures that reflect linguistic accuracy: appropriate voicing transitions and correct syllable structure.

In 2021, Namasivayam and colleagues [23] reported key measures of validity and reliability of the MSH-PWs. Their data indicate high content, construct and criterion-rated validity, as well as high reliability on measures of internal consistency and intra-rater reliability and moderate agreement on inter-rater reliability. This validation study, however, did not undertake a fine-grained analysis of individual scoring criteria (e.g., jaw range and jaw control), and the MSH-PW assessment tool was only validated on children aged 3 to 10 years with moderate-to-severe motor speech disorder. Therefore, construct validity in the form of distinguishing between the speech motor skills of TD children and SSD children and the scoring criteria involved in diagnosing impaired speech motor control have yet to be established for the MSH-PWs.

Furthermore, despite perceptual measures being used to judge articulatory control, the gold standard for evaluating speech motor control is based on instrumental analysis. Researchers have long advocated the need to combine perceptual analysis with the instrumental analysis of kinematic (i.e., the study of motion: displacement, velocity and acceleration) and acoustic measures [15,18,21]. In recent years, the enormous potential of machine learning (ML) in the support of a diagnosis of SSD has been recognized, and the use of the kinematic analysis of speech has progressed with the development of new video and motion-tracking technologies [24,25] that could potentially be used in combination with clinical tools such as the MSH-PWs. Mogren et al. [26], for example, demonstrated the ability to reliably extract measures of jaw movement using markers located on the middle upper lip, middle lower lip and chin (pogonion). While these methods provide precise motion data, they are, however, typically constrained to laboratory settings and are not yet practical for everyday clinical environments.

Computer vision-based approaches to measuring facial movements offer an objective and well-defined standard for detecting atypical speech patterns [27,28] and have demonstrated the potential for detecting facial movements associated with disorders [28,29,30]. While promising, the features used by these systems are not well grounded in the existing clinical understanding of which facial movements are involved in which aspects of speech motor control. As such, the decisions made by these systems lack explicative transparency.

This study focused on the assessment of mandibular control in young children using the MSH-PWs and reports on the development of a computer vision-based analysis system that offers objective measures of jaw movement for use in everyday clinical environments. (This study is a part of a larger study currently being undertaken that integrates automatic facial tracking data with perceptual scoring at all levels of the MSH-PWs). Motor speech control develops in a sequential yet non-linear manner, with mandibular control providing a foundation for the later refinement of labial and lingual movements [23,25,31,32,33]. Early speech production is characterized by reliance on jaw movements for oral closure, with young children showing greater mandibular displacement and less articulatory independence [34]. As development progresses, control shifts from jaw dominance to more independent and refined lip and tongue movements [32,35]. Kinematic measures have captured these developmental changes, highlighting the foundational role of the jaw in early speech motor organization [36]. Importantly, poor mandibular control has also been identified as a feature of children with SSD, including reduced range of motion of jaw movements during vowel production [26] and increased variability in movement trajectories of the jaw, indicating impaired lateral jaw stability [37]. Furthermore, there is evidence that jaw control and stability may be useful markers in determining subtypes of SSD [38].

Therefore, given the importance of assessing mandibular control in young children with speech production difficulties, we investigated whether MSH-PW mandibular control scores obtained from expert clinicians could distinguish TD children from children diagnosed with SSD. The aim was to validate the perceptual scoring of MSH-PWs as being sensitive to individual differences in speech motor skills at the level of mandibular control and identify MSH criteria that could be predictive of disordered mandibular control, relative to TD children.

We also employed a State-of-the-Art facial mesh detection and tracking algorithm [26] to extract measurements of facial movements identified as clinically salient in the assessment of speech motor control from recorded videos of children speaking words from the mandibular stage of the MSH-PWs. We aimed to evaluate how well these extracted facial movement measurements agree with perceptual scores for the jaw range and jaw control criteria of the MSH-PWs. We selected these two criteria because, when scoring jaw range and control, clinicians rely predominantly on the child’s facial movements.

This preliminary study, therefore, sought to answer the following questions:

Q1. Do the MSH-PW criteria, using expert consensus scoring, discriminate TD children from those with SSD? We predicted TD children would score more highly than SSD children in relation to the MSH-PW mandibular control criteria and that mandibular control criteria could be predictive of whether a child was TD or had an SSD.
Q2. Can kinematic measurements derived from automated facial tracking accurately predict the expert consensus perceptual scores of the MSH-PW jaw range and jaw control criteria? We expected good agreement between objective measures obtained from facial tracking and expert clinician judgements of appropriate and inappropriate jaw range and jaw control, as indicated by the predictive accuracy in logistic regression classification models, with objective measures as predictors and clinician judgements as the outcome.

## 2. Materials and Methods

### 2.1. Participants

The participants were 54 children aged between 3 years and 0 months and 3 years and 6 months who were recruited from the Perth Metropolitan area and surroundings between April 2020 and December 2024 as part of a larger ongoing study on children’s speech development. Recruitment for this larger study sought children with typical speech development; however, during the assessment process, several children with characteristics of SSD were identified. All participants had complete facial tracking and speech and language assessment data available for analysis. A total of 41 of the participants (21 male; 20 female) had typical speech development (TD), while 13 (5 male; 8 female) presented with SSD. Standardized assessments, including the GFTA-3 Sounds-in-Words subtest [39] standard score, measures of phoneme accuracy (the PPC, PVC and PCC), the Verbal Motor Production Assessment for Children (VMPAC [40]), Focal Oral Motor Control and Sequencing subtests, and parent-reported measures of intelligibility using the Intelligibility in Context Scale (ICS [41]), and clinical observations were used to determine allocation into the TD and SSD groups.

The mean age was 37.9 months for children in the TD group (range = 36 months and 6 days to 41 months and 27 days) and 37.3 months for those in the SSD group (range = 36 months and 9 days to 41 months and 23 days). There was no significant difference in age between the two groups (*t*(52) = 1.179, *p* = 0.244) nor were there significant group differences in gender (*χ*^2^(*N* = 54) *=* 0.64, *p* = 0.432). All participants were identified as having age-appropriate language and fine and gross motor development based on parent reports via the Ages & Stages Questionnaires^®^, Third Edition (ASQ^®^-3) [42] and standardized language assessments using the Clinical Evaluation of Language Fundamentals Preschool-2nd Edition, Australian and New Zealand Standardized Edition (CELF-P2 [43]), as seen in Table 1. All TD children scored within the normal range on the Goldman–Fristoe Test of Articulation, 3rd Edition (GFTA-3) Sounds-in-Words subtest (SS > 85) [40].

The SSD group comprised children who scored below average on the GFTA-3 (SS < 85, *n* = 9), or who met two or more of the following criteria (*n* = 4): scores below the 5th percentile on the VMPAC for oromotor and/or sequencing subtests; the PCC, PPC or PVC, calculated from the GFTA-3 Sounds-in-Words subtest, greater than 2 standard deviations below the total sample mean; the presence of atypical speech errors as identified by Morgan et al. [44] and Dodd et al. [45]. Group allocation was confirmed by single-case t-tests using the Singlims_ES.exe program [46], with each participant in the SSD group showing statistically significant differences between their score and the TD sample across at least two inclusion criteria measures.

Children with structural deficits (e.g., cleft lip/palate), hearing loss, English as a second language, a diagnosed language, cognitive, neuro-developmental and/or psychological disorder (e.g., cerebral palsy or autism spectrum disorder) and/or motor disorder (developmental coordination disorder, Ehlers Danlos or hypermobility) were excluded from this study. Using the GCSI 39 auto tympanometer, participants demonstrated hearing threshold levels of 20 dB or lower across each frequency of 1000 Hz, 2000 Hz and 4000 Hz, which is consistent with the ASHA Childhood Hearing Screening protocol [47]. Children who were unable to engage in assessment activities due to attentional and behavioral difficulties were also excluded from participation.

### 2.2. Procedure

#### 2.2.1. Data Collection

The CELF-P2, GFTA-3 Sounds-in-Words subtest and VMPAC assessments were completed at a home visit and in accordance with administration guidelines outlined in the respective manuals. Participants attended a laboratory at Curtin University to complete a hearing screen and the MSH-PW task. The laboratory room was selected to minimize noise and vibration and staged to be child-friendly using posters of popular children’s characters and toys (e.g., Bluey). Testing took place on weekends to further minimize background noise from campus activities close to and within the building. The average ambient noise level prior to participant arrival, using the Protech QM1589 sound level meter, was 35 dBA, which is below the 48 dB minimum sound level that Rusz et al. [48] recommend for the recording of speech.

Participants were provided with free play time to enable researchers to develop a rapport and familiarize the child with the laboratory room. For completion of the MSH-PWs, participants were seated on a custom-built chair designed for optimal head position and safety. A Blackmagic Pocket Cinema Camera 4K video camera recorded full HD (1920 × 1080p) at a frame rate of 60 frames per second. The 45 mm camera lens was placed central to the child’s chair on a Sirui SH15-CN video tripod, as detailed in Palmer et al. [49].

The researcher explained the task to the participant, stating that they would be shown pictures and asked to repeat target words after the investigator. Participants were instructed to remain seated and to continue looking at the picture throughout the task, with reminders given as needed.

A wireless Rodelink LAV microphone (RodeFilmaker Kit) was attached to the participant’s clothing, with the receiver connected to one channel of the stereo microphone input of the camera for digital audio recording. For participants who refused to wear the microphone or repeatedly handled it, the microphone was clamped to the front of the back cushion on the chair at mouth height. A Sennheiser Me66 shotgun microphone, attached to the camera and directed toward the speaker, was connected to the remaining microphone input channel and served as a second or backup audio recording if required.

The image of the MSH-PW target word was cast onto a 93 cm Samsung television directly in the child’s line of sight via an HP EliteBook laptop computer. The order of administration was randomized using an online program [50]. Participants were required to name each of the ten target pictures in response to the instruction, “say X”. All responses were videorecorded to enable facial tracking to undertake kinematic analysis and phonetic transcription. Participants were asked to repeat words when they did not produce a response or if their body and/or head movements were likely to compromise accurate facial tracking. Participants were given general feedback and encouragement for their performance and engagement in the tasks. Breaks between tasks were offered as required. The testing session lasted no more than 60 min, with variability around participant attention and the need for play breaks.

#### 2.2.2. Data Preparation

The video files from the camera were imported into Adobe Premiere Pro to identify the speech movement boundaries for each target word. These onsets and offset boundaries (timestamps) were subsequently used to mark the acoustic word boundaries for each word on text grids within the PRAAT program [51]. A program was developed using Python 3.9.6. to automate the generation of TextGrids, utilizing the PraatIO library version 6.1 [52] for efficient data processing.

##### Phonetic Transcription

Narrow phonetic transcription and phonological error analysis using the Khan-Lewis Phonological Analysis approach [53] was completed by three experienced S-LPs. Transcriptions were completed in PRAAT, with the S-LPs drawing on both the acoustic information in the speech signal and visual information from the video recordings of each participant’s target word production. Before commencing transcription, the S-LPs participated in a calibration phase. This involved collaboratively completing example case study transcriptions to establish protocols for specific PRAAT settings and the use of specific diacritics and approaches to coding variabilities (e.g., coding a final devoicing error as /p/, rather than the voiceless /b̥/). Next, each S-LP independently transcribed additional case study data, and points of difference were discussed. Following this calibration phase, participant transcriptions were randomly allocated across the three S-LPs. To ensure ongoing transcription consistency, after every eight to ten samples, one participant's sample was randomly selected for transcription by each S-LP. The inter-rater reliability of broad transcription was 86.1%. Once transcription was completed, the PPC, PVC and PCC were calculated from the broad transcription.

##### Perceptual Scoring

Consensus scoring of all five mandibular features—appropriate mandibular range, mandibular control/stability, open–close or close–open (phase) movements, voicing transitions and syllable structure—was undertaken by three certified PROMPT instructors. A binary scoring system of appropriate (1) or inappropriate (0) was assigned to each word for each feature, according to the scoring criteria detailed in the MSH-PW manual [54]. Before commencing the scoring of the study sample, the three instructors met with Ms. Deborah Hayden (DH), a co-developer of the MSH-PWs, on three occasions to discuss the scoring criteria definitions, calibrate and collaboratively assess an example case study separate from the dataset reported in this paper. Given the multidimensionality of features within the mandibular range, mandibular control and phase movements, definitions were further refined from those outlined in the MSH-PW manual to allow for consistency in scoring. Three different cases, not related to this study, were subsequently scored independently for reliability analysis. The interclass correlation coefficient, using a mixed model with absolute agreement, showed good agreement (ICCs > 0.85) between DH and the consensus scorers. After establishing inter-rater reliability, participants were independently scored in sets of five. The scores were collated by an independent research assistant, with items of difference identified. These items were resolved by reviewing the video footage and by discussion until the point of difference was resolved. This process was repeated until all participants were scored.

##### Selection of Kinematic Measurements

**Figure 1:**
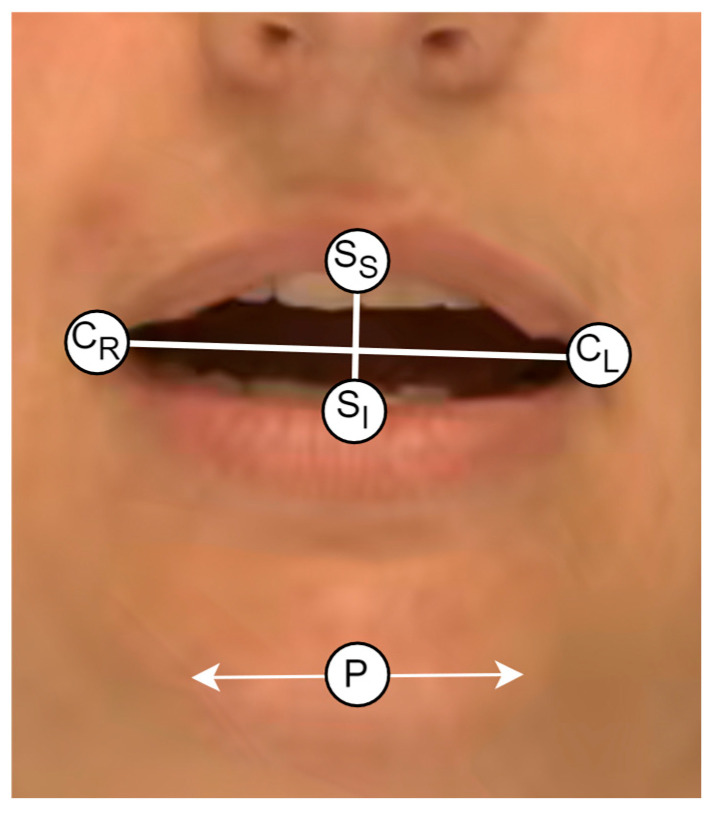
The landmarks used in the extraction of the kinematic measurements. Note: C_R_ = cheilion right, C_L_ = cheilion left, S_S_ = stomion superius, S_I_ = stomion inferius. P = pogonion.

Of the five specific criteria evaluated perceptually in Stage III (mandibular control), two facial motor movements, jaw range and jaw control/stability, were further evaluated using measures derived from computer vision-based approaches to measuring facial movements. The specific measurements included mouth-opening, which was measured as the ratio of mouth width (between cheilion) to mouth height (between stomion superius/inferius) and lateral deviation of the tip of the chin (pogonion) from the rest. These measures were adopted based on their use in the previous literature (for example, ref. [26]). The landmarks involved in these measurements are shown in Figure 1. Lines connecting the cheilion landmarks (C_R_ and C_L_) and stomion superius/inferius (S_S_ and S_I_) show the relative distances involved in calculating the mouth-opening ratio. Landmark P represents the pogonion, and the arrows indicate its recorded shift laterally from the midline. Measurements of both displacement and velocity were taken. For the lateral deviation of the pogonion, the extracted distance was normalized by a measurement of facial width derived from the upper face using distances between bilateral landmarks at the zygion, tragion and exocanthion.

**Figure 2:**
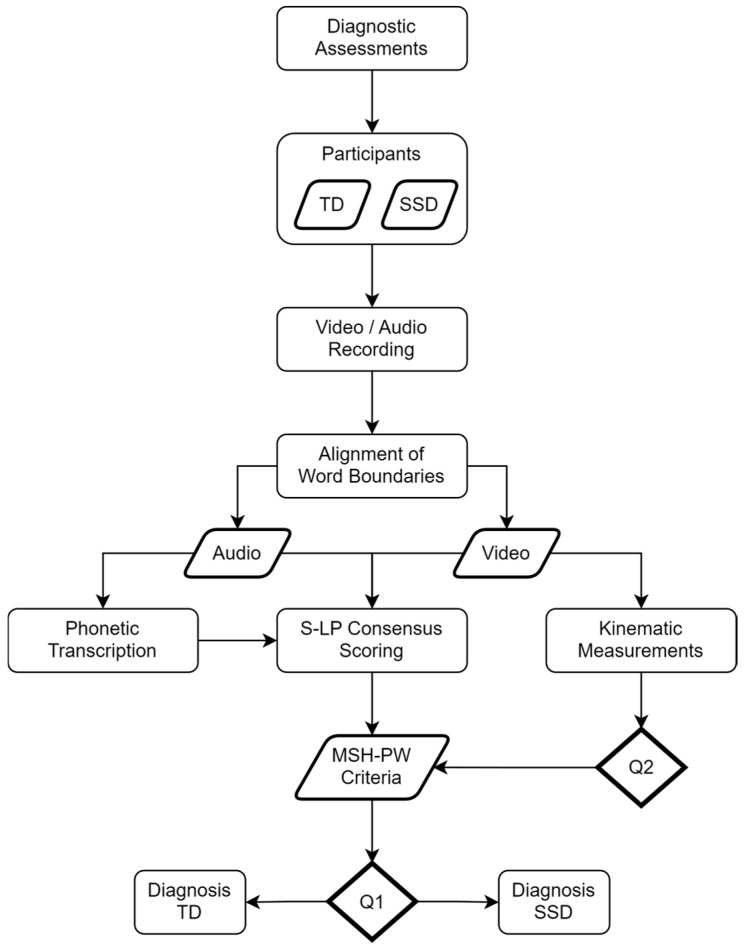
Flowchart showing data collection and processing procedures.

The flowchart in Figure 2 details the data collection and processing procedures. The analysis elements relating to Research Question 1 and 2 are shown as diamond symbols and are described in more detail in Section 2.3.

### 2.3. Data Analysis

#### 2.3.1. Analysis Related to Research Question 1

##### Perceptual Analysis

IBM SPSS Statistics (Version 29) was used to conduct independent samples t-tests or Mann–Whitney U tests (if the normality of data was violated) to compare the TD and SSD groups in the MSH-PW mandibular measures of jaw range, jaw control, phase, voicing transitions, syllable structure and total mandibular percentage score. The normality of the data distributions was assessed using the Shapiro–Wilks test (*p* > 0.05) and z-score skewness and kurtosis values within +/−1.96. The PVC, PCC and PPC obtained from the MSH-PW phonetic transcriptions were included as outcome measures. Given the preliminary nature of the study and to protect the type II error rate, we opted not to adjust the alpha level and used a 0.05 criterion for statistical significance.

##### Classification Analysis

For each of the five MSH-PW criteria, the scores were encoded as separate ten-valued feature vectors (corresponding to the binary appropriate/inappropriate scores for each of the ten words) and associated with the diagnostic label TD or SSD for training a logistic regression model (with implementation provided by the Scikit-learn Python library [55]) specific to that criterion. A meta-classifier (also a logistic regressor) was then trained on the individual outputs of the five criterion models to predict the final diagnostic label. Leave-One-Out Cross-Validation (LOOCV) was then performed to evaluate the overall scheme’s classification performance. A grid search approach was used to tune the models at both the individual criterion level and the meta-model level in terms of the ratio, strength and type of regularization used (lasso or ridge).

#### 2.3.2. Analysis Related to Research Question 2

A State-of-the-Art facial detection and tracking algorithm was used to extract time- and space-normalized measurements of facial movements from videos recorded of the 41 TD participants speaking the ten Probe Words from the mandibular stage of the MSH-PWs. The TD sample was used to maximize the data available and initially assess the prediction of scoring with a normative or TD sample without the confound of some children having an SSD. The SSD sample size was not sufficient for this analysis. Measurements were captured at a sampling rate matching the recording rate of 60 frames per second and linearly interpolated across 1000 timepoints to give sufficient granularity for spoken productions to be aligned at corresponding relative timepoints.

For each word, an expert S-LP was consulted to identify which of the facial movements, over what sub-period intervals of a word’s production, ought to best characterize it in terms of either the jaw range or the jaw control criteria of the MSH-PWs. These rules were then programmatically encoded as parameters to the classification model. The mouth-opening measurement was identified for the jaw range criterion as the sole measurement to be evaluated. For jaw control, two different measurements were used: the first derivative of mouth-opening (i.e., velocity) and the lateral deviation of the pogonion from the rest. For classification, an input feature was defined as the average of the absolute value of the z-score difference of a single participant’s range of motion in a measurement subtracted from its mean movement for the corresponding word over all other participants scored as having appropriate jaw range/control (where appropriate) in the spoken sub-period interval of the word, as shown in Table 2. For each word, LOOCV was performed using logistic regression to predict a label for jaw range/control as appropriate or inappropriate. A grid search approach was used to tune the model’s hyperparameters and maximize classification performance. This included balancing the strength and relative proportions of lasso and ridge regularization. Class weightings were also adjusted in the model to account for dataset imbalance.

## 3. Results

### 3.1. Research Question 1

#### 3.1.1. Mean Differences Between SSD and TD Groups

The mean MSH-PW mandibular subtest scores and total scores for the TD and SSD groups are presented in Table 3, along with the PVC, PCC and PPC.

The results show significantly higher scores for TD compared to SSD children for jaw range (*U*(*N* = 54) = 133.5, *p* = 0.006), voicing transitions (*U*(*N* = 54) = 132.0, *p* = 0.004) and total mandibular scores (*t*(52) = 2.524, *p* = 0.015). There was no statistically significant difference between groups on jaw control (*U*(*N* = 54) = 183.5, *p* = 0.089), the open–close or close–open phase (*U*(*N* = 54) = 197.0, *p* = 0.156) or syllable structure (*U*(*N* = 54) = 201.5, *p* = 0.085). Cohen’s d effect size was large for jaw range (0.96), voicing transitions (0.94) and total mandibular score (0.80). There was a medium Cohen’s d effect size for jaw control (0.47) and a small-to-medium effect size for phase (0.37) and syllable structure (0.35).

For phonetic accuracy using the MSH-PW mandibular word set, the TD group showed a significantly higher PVC (*U*(*N* = 54) = 125.0, *p =* 0.003), PCC (*U*(*N* = 54) *=* 137.0, *p* = 0.008) and PPC (*U*(*N* = 54) = 115.0, *p* = 0.002) than the SSD group. Cohen’s d showed a large effect size for all three measures: PVC (1.101), PCC (0.833) and PPC (1.273). These results indicate the mandibular word set provides speech samples that are sensitive to the speech production difficulties of the SSD children when compared to the TD children.

#### 3.1.2. Classification Analysis

The confusion matrix of Table 4 shows the results of classifying the perceptually scored MSH-PW criteria into the binary diagnostic labels TD or SSD. The balanced accuracy score, derived as the average of sensitivity (recall on the SSD class) and specificity (recall on the TD class), is used here to report performance due to its robustness to imbalanced datasets. Similarly, balanced precision (also known as macro-averaged precision), calculated as the average of the positive predictive value (PPV) and the negative predictive value (NPV), is used to report overall precision performance.

**Figure 3:**
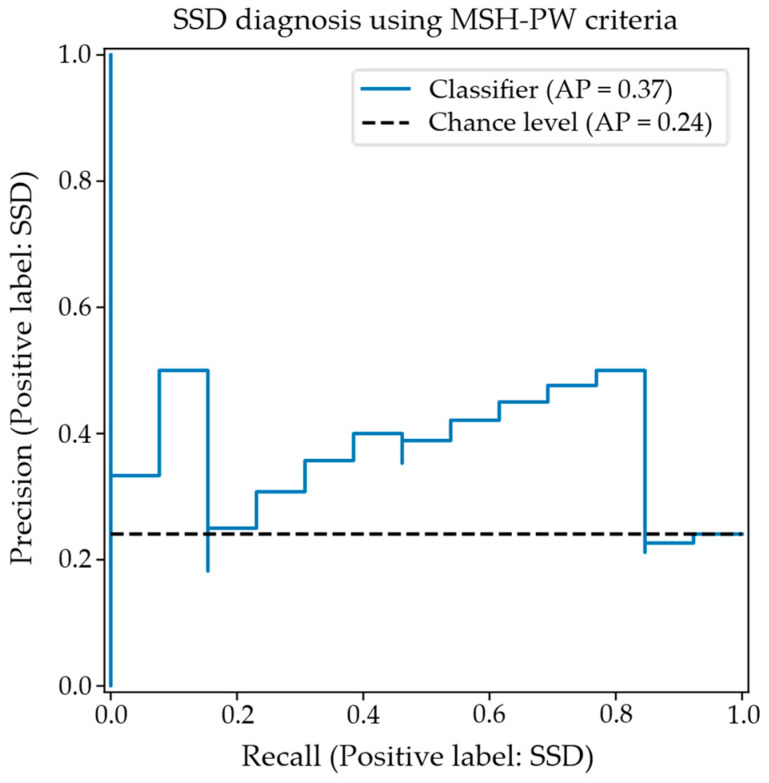
Precision–recall curve for SSD classification using MSH-PW criteria.

At an alpha of 0.01, a Monte Carlo simulation over one million runs estimated the significance threshold for the class balanced accuracy and precision statistics as approximately 0.729 and 0.661, respectively. Since the evaluated balanced accuracy and precision scores exceed these thresholds, it is concluded that the null hypothesis of no association between the mandibular perceptually scored MSH-PW criteria and the diagnostic class is rejected. The precision–recall plot of Figure 3 summarizes the classification performance for the SSD class, showing how precision/specificity varies with increasing recall/sensitivity. A typical precision–recall curve shows a relatively smooth tradeoff between recall and precision, with precision decreasing to chance levels as recall increases. The atypical profile of this plot likely indicates a lack of generalizability in the trained model.

The classification scheme was trained over the whole dataset using the hyperparameters derived through LOOCV to find the weights used by the criterion models and the meta-classifier. After normalizing, these weights (which act as coefficients for the respective features) reflect the contribution of the features in supporting the determination of the final diagnostic labels. Under the LOOCV-derived hyperparameters tuned to maximize classification performance, only the classifier trained against the jaw range scores contributed meaningfully to the final diagnostic label. Lasso regularization in the meta-classifier minimized the weights of the other criterion classifiers to zero, indicating that their contributions did not improve overall performance. Within the jaw range classifier itself, lasso regularization minimized weightings against all words except the following: “Ba”, “Eye”, “Um”, “Pam” and “Pie”. Measurements taken from the productions of the other words in the set were ignored by the jaw range classifier.

### 3.2. Research Question 2

The confusion matrix in Table 5 shows the resulting classification performance for an appropriate or inappropriate jaw range, given optimal tuning of the model’s hyperparameters via LOOCV for the evaluated sample.

**Figure 4:**
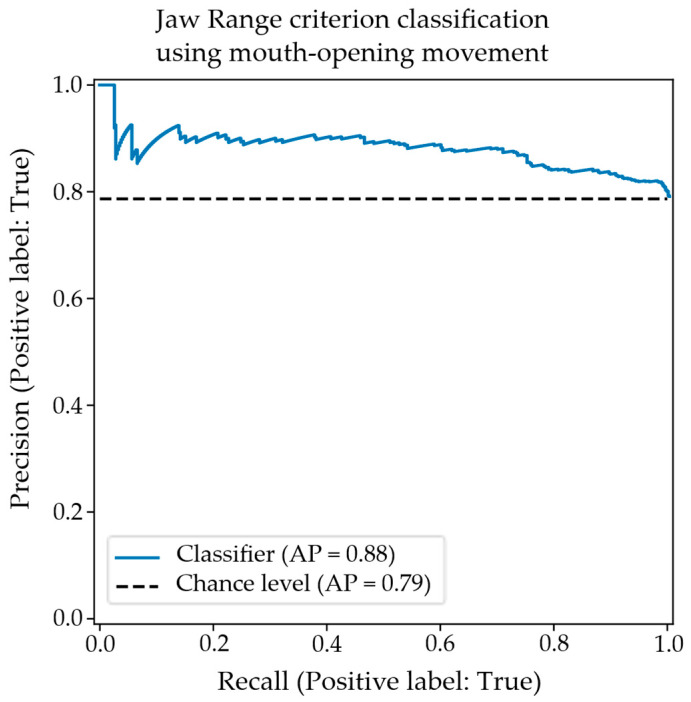
A precision–recall plot for the classification of an appropriate/inappropriate jaw range using the objective mouth-opening facial feature.

The overall balanced accuracy was calculated as 0.67, and balanced precision was calculated as 0.62. Both these statistics are higher than the respective significance thresholds of 0.583 for accuracy and 0.542 for precision derived via the Monte Carlo simulation for their occurrence by chance at alpha = 0.01. It is, therefore, concluded that the null hypothesis of no correlation between the jaw range criterion and the objective mouth-opening facial feature is rejected. The associated precision–recall plot is illustrated in Figure 4, which shows that precision stays relatively high before smoothly decreasing past approx. 75% sensitivity.

For the classification of appropriate/inappropriate jaw control using the velocity of mouth-opening, the classification performance was significant at an alpha of 0.01. The significance thresholds for balanced accuracy and precision were estimated to be 0.574 and 0.547, respectively, for the jaw control criterion. The confusion matrix and statistics for the optimally tuned model hyperparameters in Table 6 show classification performance above these thresholds, meaning that the null hypothesis of no correlation between the measurement and the jaw control score is rejected.

**Figure 5:**
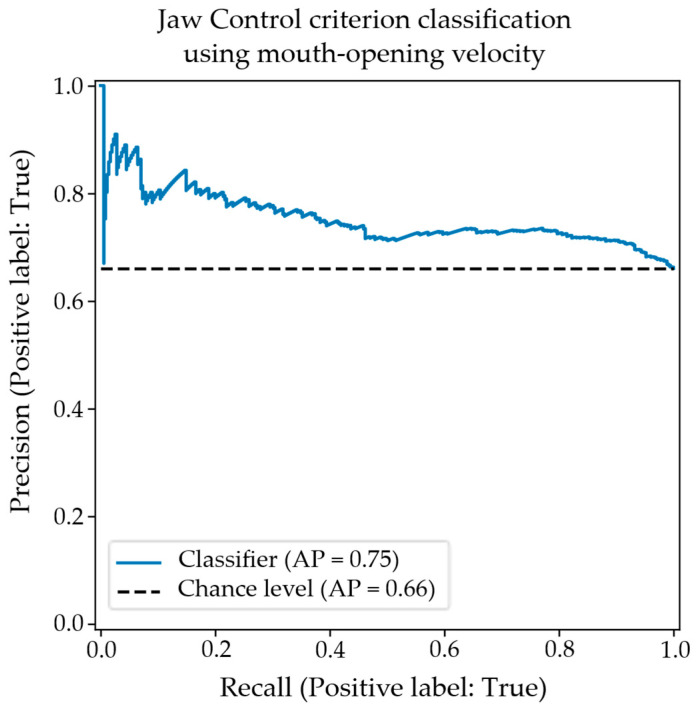
The precision–recall plot for the classification of appropriate/inappropriate jaw control using the objective “Mouth-Opening velocity” facial feature.

The associated precision–recall plot is shown in Figure 5. Precision starts high before plateauing around 50% sensitivity and tailing off from 80% sensitivity.

**Figure 6:**
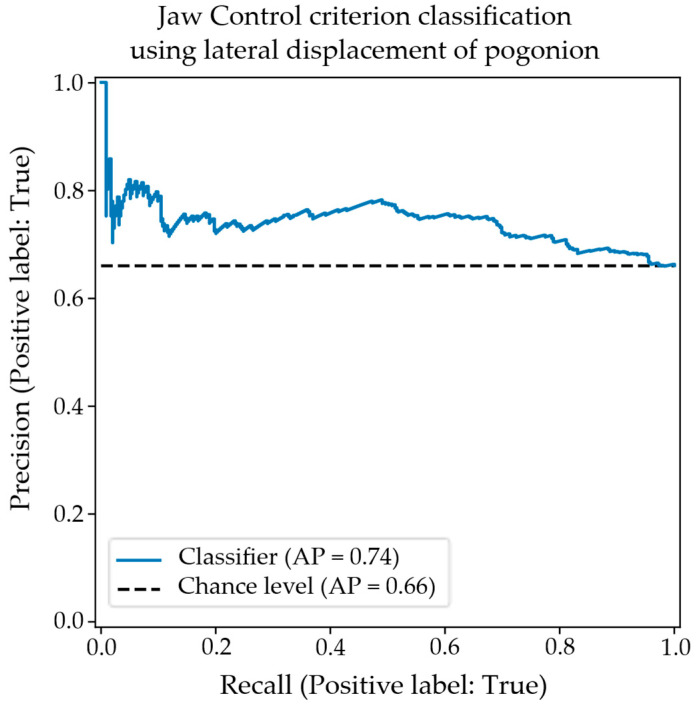
The precision–recall plot for the classification of appropriate/inappropriate jaw control using the objective measurement of pogonion lateral displacement.

The confusion matrix showing results for the classification of jaw control using the objective measurement of the lateral displacement of the pogonion is shown in Table 7. Again, the results for balanced accuracy and precision are significant at alpha = 0.01; however, the relative incidence of type I and type II errors is reversed. This potentially indicates that the features are characterizing different aspects of the data. The associated precision–recall plot is shown in Figure 6. The classification performance tracks similarly to that obtained using the velocity of the mouth-opening feature.

**Figure 7:**
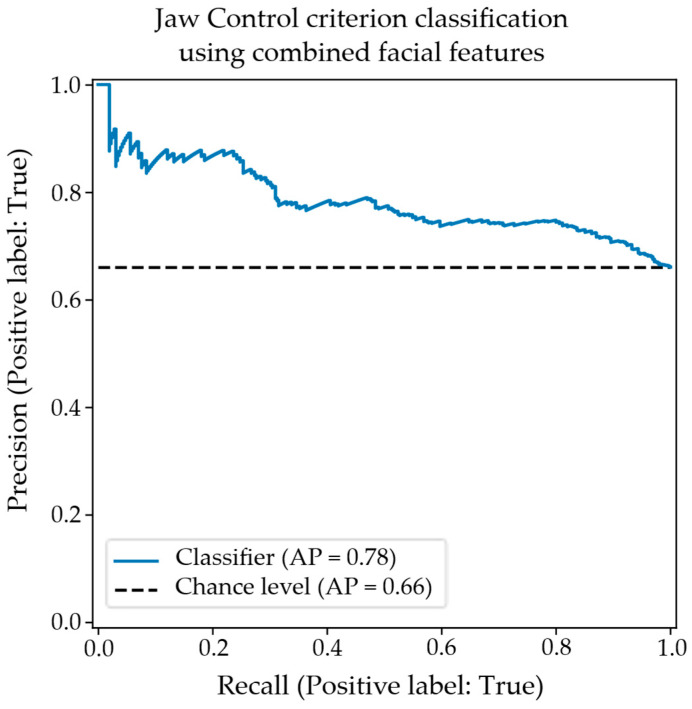
The precision–recall plot for the classification of appropriate/inappropriate jaw control using the combined objective facial features.

Finally, the confusion matrix in Table 8 shows the classification performance when combining both the mouth-opening velocity and the lateral displacement of pogonion features. In this case, it was possible to tune the model’s hyperparameters to improve classification performance over that obtained by either feature alone, offering evidence of the multidimensional nature of the jaw control criterion. The associated precision–recall plot shown in Figure 7 shows improved precision at all levels of sensitivity.

## 4. Discussion

In this paper, we report on a preliminary study aimed at exploring the potential of the MSH-PWs to discriminate the speech of children with SSD from TD speech through evaluation of the Stage III mandibular control word set. We first aimed to determine whether the observations of speech pathologists on the five MSH-PW mandibular control features of appropriate mandibular range, mandibular control/stability, open–close or close–open movements, voicing transitions and syllable structure could accurately classify children with TD speech from those with an SSD. Secondly, we evaluated the agreement between the subjective visual observations of the consensus scores completed by three S-LPs derived from kinematic measurements, which were extracted from a State-of-the-Art facial mesh detection and tracking algorithm. These research questions were selected to inform the development of norms for the MSH-PWs for assessing impaired speech motor control in children and to evaluate the feasibility of supporting S-LPs with objectively acquired measurements of motor speech control, framed within the MSH-PW scoring criteria. Each research aim is discussed in turn.

### 4.1. Classification of Children Based on Perceptual Scoring

The results of this study found there were significant differences in MSH-PW jaw range, voicing transitions and mandibular total scores between children with TD speech and those with SSD when scored perceptually by S-LPs. Discrimination analysis indicates a significant correlation between perceptually scored MSH-PW mandibular criteria and diagnostic class, as determined by a battery of diagnostic tools used in current clinical practice. This suggests that the perceptual scoring of the MSH-PW mandibular subtest can discriminate between children with TD speech and those with SSD, with potential for the MSH-PWs to be used by S-LPs in diagnosing impaired speech motor control.

The finding of no significant difference in jaw control contrasts with the existing literature that has established the significance of jaw control and stability in speech sound production [25,56,57,58,59]. Jaw control is considered a foundational aspect of speech development, with research indicating that its maturation follows a protracted trajectory. In early development, jaw movement velocities are slower and more variable in young children [35]. By around 6–8 years of age, these movements become more refined and less variable [26,36], reflecting the gradual development of stable control. Wilson and Nip [60] highlighted the importance of jaw control and stability in supporting lip and tongue movements, noting its involvement in nearly all articulatory positions.. Poor jaw control has been identified as a feature of SSD. For example, Mogren and colleagues found children with SSD had larger lateral jaw movements than children with TD speech [26]. Similarly, Terband et al. [37] identified clear deviances in lateral jaw movement within their SSD group compared to their sample of TD participants. The reasons for the contrast in these findings with this current study were considered.

Firstly, previous studies examining motor control tend to feature participants with clearly identified motor speech disorders (e.g., ref. [59]), including childhood apraxia of speech (e.g., ref. [61]) and/or may differentiate between various subtypes of SSD. For example, Terband et al. [37] differentiated children with phonetic articulation disorder, phonological disorder and childhood apraxia of speech. Participants in this study reflected real-world referral patterns, and those in the SSD group of this current study had not been previously identified as having an SSD before their participation. Some parents expressed uncertainty over whether their child’s speech was developing within age expectations; however, difficulties accessing developmental screening had impeded prior access to speech pathology services. Information from the WA Department of Health, Child and Adolescent Health Services indicates that between July 2019 and June 2020, only 44% of 12-month-old children and 30.2% of 2-year-old children were seen for their universal health check, which includes a developmental screen. The COVID-19 pandemic was noted to have further reduced access to this service in the preceding years [62]. As such, it is possible that participants in the SSD group demonstrated milder SSD features and/or that children within this group primarily had difficulties at the phonological level rather than motor-based difficulties. This suggestion is supported by Terband et al.’s finding that a participant with a phonetic articulation disorder had “very normal values” on lateral jaw movement. As such, it is plausible that the results of this current study may be reflective of the characteristics of participants in the SSD group. Further analysis of children with diagnosed motor speech difficulties may yield classification differences across a wider range of MSH-PW criteria.

Secondly, the mandibular word set items were intentionally selected to include only bilabial consonants and low vowels, with targets achieved through open–close (e.g., um), close–open (e.g., ba), close–open–close (e.g., map) and close–open–close–open (e.g., papa) jaw movements. With the vowel target determining jaw height, differences in the PVC and PPC between the TD and SSD groups indicate there are differences in speech production accuracy from an auditory perceptual perspective that may not be evident in perceptual movement analysis over the ten mandibular target items. Furthermore, difficulties in jaw control, specifically, may not be evident until motor complexity increases in the higher MSH-PW stages. Further investigations are required to determine the impact on jaw control as young children are required to integrate jaw stability with labial–facial and lingual movements and sequencing these movements in multisyllabic and phrase-level speech.

Finally, perceptual scoring of the MSH-PWs involves determining whether the child’s production meets the criterion of appropriate or inappropriate. This binary scoring system may have obscured mild features of SSD in the SSD group participants. Further research may consider whether an ordinal scoring system enhances the identification of children with mild features of SSD or subclinical features that may indicate speech delays.

### 4.2. Agreement Between Perceptual Scoring and Kinematic Measures

Our second research question sought to explore the agreement between the perceptual scores of jaw range with the kinematic measure labeled mouth-opening and jaw control with two kinematic measures labeled mouth-opening velocity and lateral movement of the pogonion. We found good agreement for both jaw range and jaw control.

The rating of jaw range required consensus raters to make a binary judgement of appropriate or inappropriate for age, where a judgement of inappropriate was given for movements considered restricted or overextended, as defined for each vowel height position for each word in the MSH-PW scoring manual. Our preliminary results suggest the objective measure of mouth-opening could be used to support speech pathologists in their assessment of jaw range.

It is proposed that the agreement between the consensus raters with the mouth-opening measurement resulted from their already established internal representation of jaw height and that this representation was aligned with the objective kinematic measures, resulting in good agreement. This proposal is based on the knowledge that the consensus scorers are familiar with the vowel quadrilateral that describes jaw and tongue positions, as well as the established body of literature that specifies that jaw height adjustments contribute to the production of vowels [63,64]. It is, therefore, conceivable that the raters utilized this knowledge, along with their experience, to inform their decision-making. That is, when a child produced a vowel error, the associated jaw height position could be evaluated as too high or too low with respect to the intended target.

Similarly, there was good agreement between the consensus scores and kinematic measures for jaw control. The rating of jaw control required the consensus raters to make a binary judgement of appropriate or inappropriate for age, based on velocity and the midline or anterior–posterior stability of the jaw. The finding that the combined measures showed greater agreement than the individual measure is likely reflective of the multidimensionality of the jaw control criterion and jaw control movements in general. Multidimensionality is an essential feature of jaw movement and the integration and balancing of vertical, lateral and rotational movements with precise timing and velocity to enable speakers to adapt to the acoustic and articulatory demands of different phonemes and provide a dynamic scaffold for tongue and lip movements [58,59,61]. As outlined, the rating for jaw control is based on several criteria reflective of controlled, smooth speech movements within the vertical plane. The velocity of mouth-opening and lateral displacement of the pogonion are both key metrics of jaw control, and the interplay of each movement contributes to the production of fluent, intelligible speech.

Agreement between consensus scorers and kinematic measurements derived from automated facial tracking was likely aided by the high level of experience the consensus scorers had in the assessment of speech motor control. Further research should explore the level of agreement in ratings of jaw range and jaw control with S-LPs who have less experience in the assessment of speech motor control and determine whether kinematic measures can support the clinical judgements of S-LPs of differing levels of experience when scoring jaw range and jaw control criteria of the MSH-PWs.

### 4.3. Clinical Application

Perceptual, single-word speech assessments, such as the GFTA-3, are a critical component in the assessment of children’s speech and the diagnosis of SSD [12,13], providing timely and convenient measures of speech development and accuracy. As highlighted, there are limitations with the current perceptual assessments, including their focus on identifying phonological deficits rather than also assessing underlying speech motor control difficulties [19]. The MSH-PWs were designed to measure inappropriate speech motor control through the perceptual, visual and auditory assessment of single-word production. The findings of this preliminary study of the Stage III mandibular control level of the MSH-PWs indicate the assessment tool can identify perceptual differences in the appropriateness of jaw range and voicing transitions between children with TD speech compared to those with SSD and support further research into the additional levels of the MSH-PW: Stage IV: labial–facial control, Stage V: lingual control and Stage VI: sequenced movements. The generation of normative data for children’s performance at these levels of speech motor control, along with the MSH-PW total scores, would be beneficial.

## 5. Limitations

Data were analyzed from a small sample of children comprising 41 TD participants and 13 SSD participants. All children were within the age range of 3;0 to 3;6 years. This small, limited age sample limits generalization of the findings to a larger population and those younger or older than this target age. Children aged between 3;0 and 3;6 years frequently present with speech sound errors (e.g., ref. [65]), and it is possible that participants in the TD sample scored within age expectations on standardized assessments at the time of their participation in the study but may be identified with SSD as they get older and error patterns that are currently developmentally appropriate persist [45]. Similarly, the SSD group comprised children who had not previously been identified as having SSD, suggesting their SSD features may have been less severe and are not a broad representation of the severity of SSD in children seeking speech pathology assessment and intervention. Additionally, the SSD group was not diagnosed according to subtype using a classification framework (e.g., phonological disorder and childhood apraxia of speech) [66]. The SSD children, as a group, were significantly lower than the TD controls on VMPAC scores, indicating poorer oromotor and sequencing skills, which suggests some speech motor involvement within the SSD group. Future research with a larger sample size is needed to investigate the role of the MSH-PW word sets in differentially diagnosing subtypes of SSD.

Data analysis was also restricted to the MSH-PW mandibular word set. These 10 words contain a limited set of consonants (e.g., m, p and b), which may not be sufficient to accurately perceive differences in jaw control, phase and syllable structure. Work is in progress to analyze the remaining stages (thirty words and four phrases) of the MSH-PWs.

Finally, this study sought to discover if it was possible to associate three different facial measurements extracted from recorded videos with the mandibular range and control criteria of the MSH-PWs. While these measurements have been used in previous studies (e.g., [26]), the accuracy/precision of their extraction for use in this study was not evaluated. Irrespective of this, strong (though preliminary) positive correlations were found between the measurements and the clinician-scored criteria. Assessing how accurately these measurements record salient facial movement is the focus of a study currently in progress. The results of that study will allow the measurement process to be refined, likely improving the extracted characterization of facial movements necessary to distinguish between disordered and typically developing mandibular control. Further investigation of facial measurements other than those tested herein is also warranted, especially given the likely multidimensional nature of the MSH-PW criteria.

## 6. Conclusions

In this study, children with speech sound disorder (SSD) showed significant differences compared to children with typically developing speech in measures of jaw range, voicing transitions and total mandibular score, as scored on the MSH-PWs. In 3-year-old children, inappropriate jaw range and voicing transitions may serve as relevant markers of an underlying deficit in the mandibular component of speech motor control that could impact articulation accuracy and limit a child’s speech intelligibility. Future work is focused on undertaking analyses of the increasing phonetic and motor demands of Stages IV, V and VI of the MSH-PWs. The findings of these analyses will further inform validation of the MSH-PWs and the potential of the MSH-PWs to identify issues with motor speech control in children with SSD [23].

Further, the good agreement between consensus raters and the objective measures of speech-related mouth movements, obtained using a State-of-the-Art facial mesh detection and tracking algorithm, suggests the objective measures of motor speech control are clinically feasible. Future investigation will explore the relationships between other extracted facial measurements and criteria of the MSH-PWs in addition to those reported in this paper. We will utilize a data-driven approach using extracted measurements from videos and evaluate their relationship to diagnosis.

## Figures and Tables

**Table 1:** Mean (*SD*) participant characteristics for 3-year-old typically developing and speech sound-disordered children.

Participant Characteristics	TD (*n* = 41)	SSD (*n* = 13)
Age (Months)	37.90 (1.61)	37.31 (1.65)
ASQ-3		
Communication ^a^	56.39 (5.02)	52.78 (6.18)
Personal Social ^a^	54.03 (5.58)	52.22 (4.41)
Problem Solving ^a^	56.67 (5.61)	56.67 (4.33)
Fine Motor ^a^	49.17 (10.79)	53.33 (6.12)
Gross Motor ^a^	55.83 (5.79)	55.00 (4.33)
CELF-P2		
Core Language SS ^b^	106.97 (9.32)	101.45 (10.40)
Core Language PR ^b^	65.15 (20.59)	58.00 (28.77)
GFTA-3		
Sounds-in-Words SS	**103.48** (8.96)	**83.92** (7.24)
Sounds-in-Words PR	**57.83** (20.61)	**16.54** (12.69)
PCC	**80.81** (9.85)	53.58 (9.05)
PVC	**99.20** (1.52)	**94.25** (6.24)
PPC	**87.10** (6.12)	**69.03** (4.55)
VMPAC		
Focal Oral Motor ^c^	**61.53** (14.46)	**40.29** (13.28)
Sequencing ^c^	**52.36** (14.68)	**32.00** (15.00)
ICS (Total Score)	**23.37** (2.38)	**21.83** (1.03)

Note: SS = standard score; PR = percentile rank. ASQ-3 = Ages & Stages Questionnaires^®^, Third Edition; CELF-P2 = Clinical Evaluation of Language Fundamentals Preschool Australian and New Zealand Standardized 2nd Edition; GFTA-3 = Goldman–Fristoe Test of Articulation, 3rd Edition; VMPAC = Verbal Motor Production Assessment for Children. PCC = percentage of consonants correct. PVC = percentage of vowels correct. PPC = percentage of phonemes correct. The focal oral motor and sequency subtests are percentage scores. Means in bold indicate the difference between the TD and SSD groups was statistically significant (*p* < 0.05). ^a^ *n* = 36 and *n* = 9 for TD and SSD, respectively, due to missing data. ^b^ *n* = 39 and *n* = 11 for TD and SSD, respectively, due to missing data. ^c^ *n* = 27 and *n* = 11 for TD and SSD, respectively, due to missing data.

**Table 2:** Measurement sub-period intervals for MSH-PW criteria classification.

Probe Word	MSH-PW Criteria	Sub-Period Interval (%)
Ba	Jaw Range	[20, 60]
	Jaw Control	[10, 40]
Eye	Jaw Range	[20, 60]
	Jaw Control	[10, 40]
Map	Jaw Range	[10, 50]
	Jaw Control	[5, 70]
Um	Jaw Range	[0, 70]
	Jaw Control	[0, 70]
Ham	Jaw Range	[10, 60]
	Jaw Control	[10, 60]
Papa	Jaw Range	[10, 40], [45, 80]
	Jaw Control	[10, 40], [45, 80]
Bob	Jaw Range	[10, 50]
	Jaw Control	[10, 50]
Pam	Jaw Range	[10, 60]
	Jaw Control	[10, 60]
Pup	Jaw Range	[10, 50]
	Jaw Control	[10, 50]
Pie	Jaw Range	[15, 50]
	Jaw Control	[15, 50]

**Table 3:** Means, standard deviations and Cohen’s d for MSH-PW mandibular scores for typically developing and speech sound-disordered children.

	TD	SSD	d (95% CI)
Jaw Range	**8.29** (1.79)	**6.46** (2.22)	0.96 (0.31–1.61)
Jaw Control	6.95 (2.96)	5.38 (2.84)	0.53 (−0.10–1.16)
Phase	6.54 (3.29)	5.23 (2.74)	0.41 (−0.22–1.04)
Voicing Transitions	**9.07** (1.17)	**7.92** (1.38)	0.94 (0.28–1.59)
Syllable Structure	9.76 (0.54)	9.54 (0.52)	0.41 (−0.22–1.03)
Mandibular Percent Total	**81.22** (15.33)	**69.08** (14.37)	0.80 (0.16–1.44)
PVC	**91.23** (8.30)	**79.50** (16.17)	1.10 (0.44–1.75)
PCC	**89.93** (10.22)	**80.18** (13.84)	0.83 (0.18–1.47)
PPC	**89.93** (7.31)	**79.75** (9.93)	1.27 (0.60- 1.94)

Note: The group means in bold are significantly different.

**Table 4:** Confusion matrix and statistics for the classification of TD and SSD participants given expert consensus perceptual scoring of MSH-PWs.

		Predicted			
		TD	SSD	Recall	Precision
True	TD	30	11	0.73	0.94
	SSD	2	11	0.85	0.50
		Bal. Acc./Prec.		0.79	0.72

**Table 5:** The confusion matrix and statistics for the classification of an appropriate/inappropriate jaw range using the objectively measured “Mouth-Opening” facial feature.

		Predicted			
		Inapp.	App.	Recall	Precision
True	Inapp.	71	45	0.61	0.38
	App.	117	307	0.72	0.87
		Bal. Acc./Prec.		0.67	0.62

**Table 6:** The confusion matrix and statistics for the classification of appropriate/inappropriate jaw control using the objectively measured “Mouth-Opening velocity” facial feature.

		Predicted			
		Inapp.	App.	Recall	Precision
True	Inapp.	85	100	0.46	0.50
	App.	84	271	0.76	0.73
		Bal. Acc./Prec.		0.61	0.62

**Table 7:** The confusion matrix and statistics for the classification of appropriate/inappropriate jaw control using the objective measurement of the lateral displacement of the pogonion.

		Predicted			
		Inapp.	App.	Recall	Precision
True	Inapp.	109	76	0.59	0.46
	App.	127	228	0.64	0.75
		Bal. Acc./Prec.		0.62	0.61

**Table 8:** The confusion matrix and statistics for the classification of appropriate/inappropriate jaw control using the combined objective facial features.

		Predicted			
		Inapp.	App.	Recall	Precision
True	Inapp.	88	97	0.48	0.55
	App.	71	284	0.80	0.75
		Bal. Acc./Prec.		0.64	0.65

## Data Availability

Data are available subject to the ethical considerations of this study.
